# Allogenic Vγ9Vδ2 T cell as new potential immunotherapy drug for solid tumor: a case study for cholangiocarcinoma

**DOI:** 10.1186/s40425-019-0501-8

**Published:** 2019-02-08

**Authors:** Mohammed Alnaggar, Yan Xu, Jingxia Li, Junyi He, Jibing Chen, Man Li, Qingling Wu, Li Lin, Yingqing Liang, Xiaohua Wang, Jiawei Li, Yi Hu, Yan Chen, Kecheng Xu, Yangzhe Wu, Zhinan Yin

**Affiliations:** 1 Biomedical Translational Research Institute and The First Affiliated Hospital, Jinan University, 601 W Ave Huangpu, Guangzhou, 510632 Guangdong People’s Republic of China; 2 Department of Oncology, Fuda Cancer Hospital, School of Medicine, Jinan University, Guangzhou, 510665 Guangdong People’s Republic of China; 3 Department of Biological Treatment Center, Fuda Cancer Hospital, School of Medicine, Jinan University, Guangzhou, 510665 Guangdong People’s Republic of China

**Keywords:** Gamma delta (γδ) T cells, Immunotherapy, Cholangiocarcinoma, Clinical trial

## Abstract

**Background:**

Cholangiocarcinoma (CCA) is a highly aggressive and fatal tumor. CCA occurs in the epithelial cells of bile ducts. Due to increasing incidences, CCA accounts for 3% of all gastrointestinal malignancies. In addition to comprehensive treatments for cancer, such as surgery, chemotherapy, and radiotherapy, during the past few years, cellular immunotherapy has played an increasingly important role. As a result of our research, we have discovered the γδ T cell-based immunotherapy for CCA.

**Case presentation:**

A 30-year-old male (https://www.clinicaltrials.gov/ ID: NCT02425735) was diagnosed with recurrent mediastinal lymph node metastasis after liver transplantation because of Cholangiocarcinoma (stage IV). In the course of his therapy sessions, he only received allogenic γδ T cell immunotherapy from August, 2017 through February, 2018 (8 infusions in total). γδ T cells were expanded from peripheral blood mononuclear cells (PBMCs) of healthy donor, and ~ 4 × 10^8^ cells were adoptive transferred to the patient.

**Conclusion:**

In the above case report of the Cholangiocarcinoma (stage IV) patient who had received liver transplantation and afterward was diagnosed with recurrent mediastinal lymph node metastasis, we clinically proved that allogenic γδ T cell treatment had no adverse effects. We observed that allogenic γδ T cell treatments positively regulated peripheral immune functions of the patient, depleted tumor activity, improved quality of life, and prolonged his life span. After 8 γδ T cell treatments, the size of lymph nodes was remarkably reduced with activity depletion. This clinical work suggested that allogenic γδ T cell immunotherapy could be developed into a promising therapy drug for CCA.

**Electronic supplementary material:**

The online version of this article (10.1186/s40425-019-0501-8) contains supplementary material, which is available to authorized users.

## Introduction

Cholangiocarcinoma (CCA) is the most common malignancy of the biliary tree; it may cause fatal consequences in a short period of time [1–3]. Currently, the pathogenesis of this disease has not yet been clearly defined, although high-risk factors, such as Primary Sclerosing Cholangitis (PSC), fibrous polycystic liver, intrahepatic bile duct stones, parasitic infections, hepatitis B virus infection, chemical carcinogen exposure, diabetes, and smoking were reported to be probably related to CCA incidences [4, 5]. CCA is highly aggressive and metastatic; statistics have shown an approximate median survival of 24 months [6, 7].

For recurrent CCA, however, the median survival is only 9 months, and the five-year survival is less than 5% [8].

Because of poor efficacy results and prognoses of existing treatments for malignant cancer, the most up-to-date treatments are continually being researched, or under clinical trials. Among new developing therapeutics, immune cell therapy is emerging as an important alterative for malignant cancer treatment, particularly after the success of CD19 CAR-T [9, 10]. However, for all existing adoptive immune cell therapy, autologous T cells were applied because of MHC restriction. Until present, there have been no reports concerning allogenic T cell applications regarding clinical safety or efficacy. As for γδ T cells, all previous reported works only focused on autologous cells (in vitro or in vivo expansion strategy) as well [11–20].

In this report, we applied allogenic γδ T cells (Vγ9Vδ2 subsets) as a new type of immune cell therapy to treat CCA. To our knowledge, our work provided the first paradigm on using allogenic γδ T cells to treat cancer. Previously, literatures have demonstrated that γδ T cells are the “first line of defense” as an antitumor effector cell [21, 22], for instance, γδ T cells provide an early source of IFN-γ in the tumor microenvironment [23]. Unlike αβ T cells, γδ T cells recognize antigens in a non-MHC restriction manner. Molecules like LFA, NKG2D, CD16, and others play key roles in γδ T cell recognition and killing of cancer cells. Altogether, γδ T cells could be a promising candidate for cancer immunotherapy [24–26].

In addition, for the first time via this clinical trial study for CCA, we discovered evidence that allogenic γδ T cells in immunotherapy are clinically safe and risk-free. In this case, the patient only received allogenic γδ T cell treatments. We did not observe any side effects after cell infusions, and more strikingly, peritoneal lymph node metastasis was depleted. Currently, the patient’s condition is completely released and stable. The Regional Ethics Committee of Guangzhou Fuda Cancer Hospital approved the study protocol (Approval ID 2017–02). Written informed consent was obtained from the participant, in accordance with the *Declaration of Helsinki*. And ClinicalTrials.gov ID: NCT02425735.

## Case report and methods

A 30-year-old man was diagnosed as Cholangiocarcinoma with mediastinal lymph node metastasis stage IV. In July 2013, he received treatment at a local hospital for Crohn’s disease. In Nov. 2014, he received a liver transplantation; a huge tumor at hepatic portal was intraoperatively resected. The postoperative pathology report revealed a liver and hepatic portal poorly-differentiated adenocarcinoma with unresectable Cholangiocarcinoma metastasized to lymph nodes.

The MRI scan performed on Feb. 24th, 2015 showed a lesion in patient’s liver, therefore, he received lymph node resection on Apr. 13th, 2015. From Jun. 13th, 2015 to Aug. 14th, 2015, the patient received radiotherapy for hepatic portal and the area adjacent to inferior vena cava, with a total dosage of 45Gy. Afterward, the patient did not receive any further anti-cancer treatments, except follow-up visits. The PET/CT collected on Apr. 15th, 2016, showed lesions in mediastinum and liver. On Jun. 29th, 2016, the patient came to the Fuda Cancer Hospital. Firstly, aspiration biopsy was conducted and 10 I^125^ was seeded into the mediastinal tumor. On June 2017, when the patient came back the Fuda Cancer Hospital for follow-up check-up, biopsy result showed recurrent abdominal lymph node metastasis by experts’ consultation, therefore starting from June 2017, the patient only received γδ T cell immunotherapy to control his lesions, and the first γδ T cell infusion was scheduled on August 2017.

### Immunotherapy

**Fig. 1:**
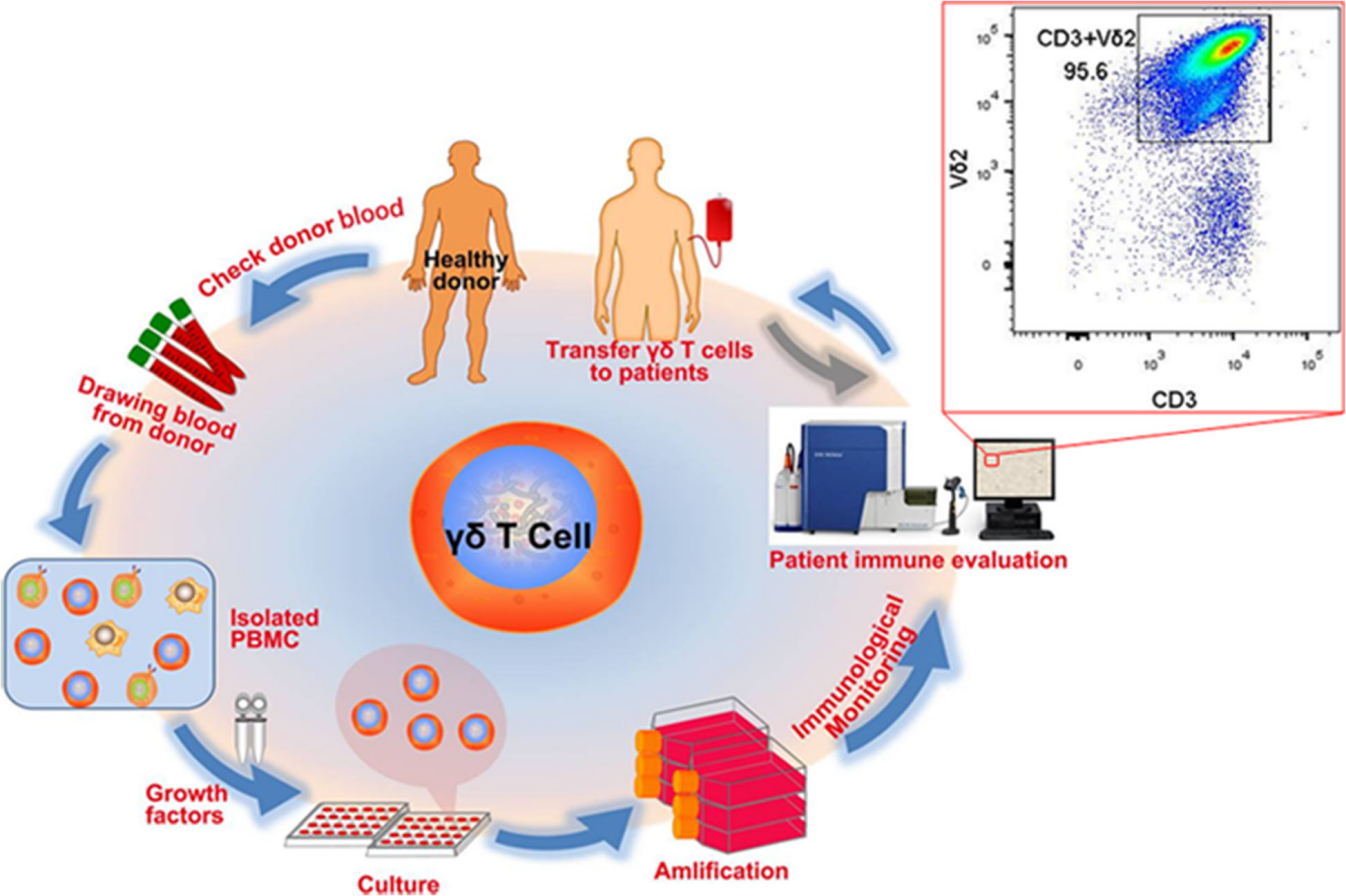
A sketch diagram describing immunotherapy from allogenic γδ T cell expansion to infusion: check donor blood (infection diseases), draw peripheral blood (100 ml) from healthy volunteer, isolate PBMC, cell culture and amplification, quality controlling and finally adoptive transfer γδ T cell to patient. The allogenic γδ T cells expanded in vitro were quality-controlled using immunofluorescence labeling and flow cytometry analysis. Quality controlling was performed before every cycle’s intravenous infusions. In our work, patient immune cell function was also analyzed before and after γδ T cell treatments by analyzing peripheral immunophenotypes using flow cytometry

**Fig. 2:**
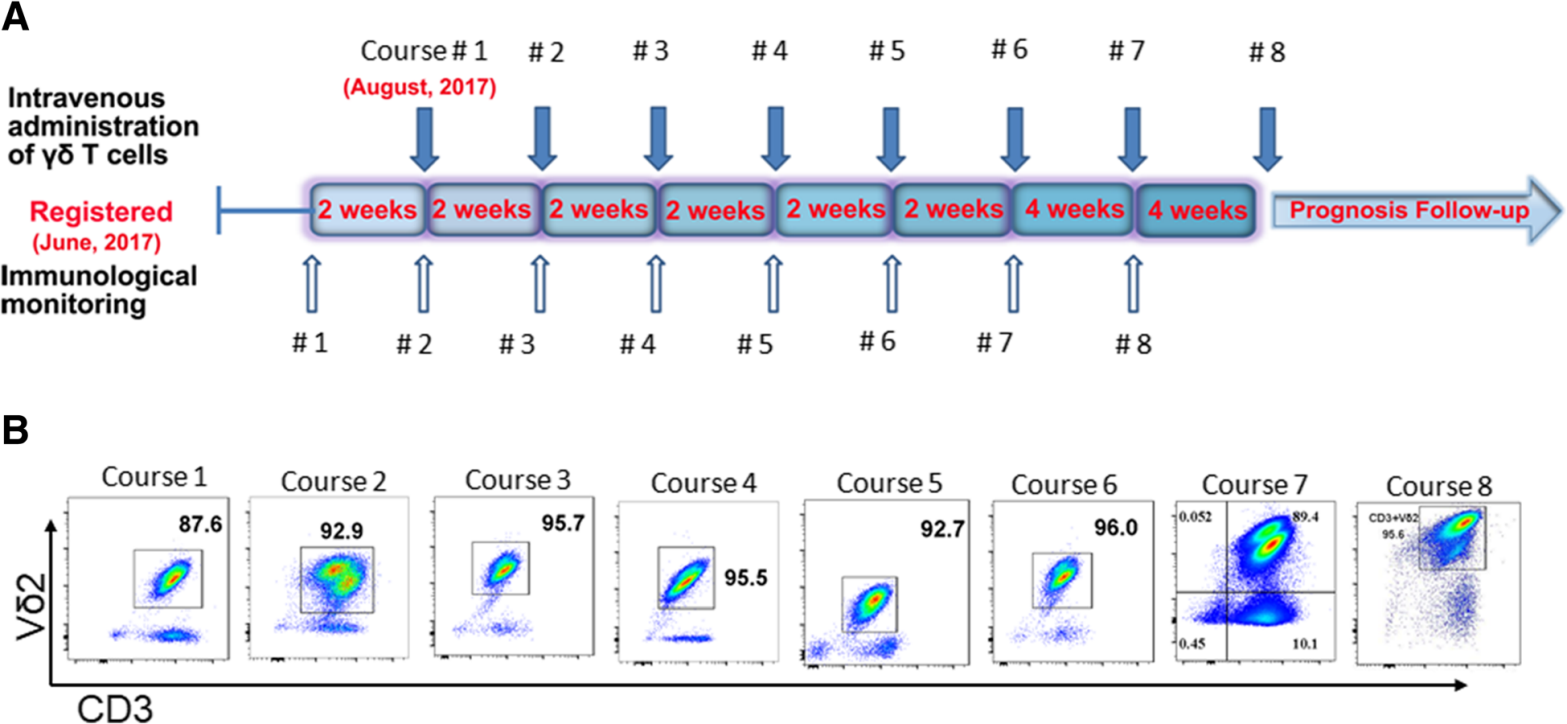
**a** Schematic diagram on schedules of γδ T cell treatments and immunophenotypes monitoring. Patient was enrolled in on June 2017, and received cell treatments starting from August 2017. The patient received 8 treatment courses (3 infusions per treatment course infused within 2 days) of γδ T cell treatments from August, 2017 through February 2018. As (**a**) showing, infusion was performed every 2 weeks for first six infusions, and then 4 weeks for last two infusions. Moreover, before and after γδ T cell treatments, immunophenotyping of the patient was checked up each time. **b** Purity phenotype of infused allogenic Vγ9Vδ2 T cells for each treatment course. It shows > 85% Vδ2 T cells in CD3+ T lymphocytes were intravenously infused. As for phenotypes of infused Vδ2 T cells and non-Vδ2 T cells were attached in Additional file 1: Figures S1 and S2

100 ml of blood was donated by a donor who had passed a health examination that included a check for infectious diseases. Following this procedure, a cell culture formula, which we developed **(**patent pending**)** that included zoledronic acid and a variety of interleukin was applied specifically to expand Vγ9Vδ2 T cells in vitro (culture media components and mechanism will be discussed in detail in our preparing article). With this formula, we can generally obtain 300–400 million of Vδ2 T cells at ~ 12 days. Figure 1 shows a brief illustration on cell expansion and cell quality control as well as cell reinfusion, and Fig. 2 indicates schedules of γδ T cell treatments and immunophenotypes monitoring (Additional file 1: Figure S1 and S2).

### Immunophenotype evaluation

5 mL of peripheral blood was extracted from the patient each time, 1–3 days before receiving Vδ2 T cell treatment. Peripheral blood monocyte cells (PBMC) were isolated using the Ficoll recipe. Then immunofluorescence labeled cells were analyzed using flow cytometry (FACSanto™ II; BD Biosciences, San Jose, CA, USA). The analyzed immune cells mainly included T lymphocytes, NK cells, and γδ T cells.

### Tumor monitoring by MRI imaging and follow-up

During Vδ2 T cell treatment, tumor was routinely evaluated by using MRI imaging to monitor tumor size/area changes by the largest transverse diameter, particularly before and after treatment. The patient received plain and enhanced MRI 2 weeks before treatment, and then scanned periodically at the 3rd and 6th months after treatment.

## Results

**Fig. 3:**
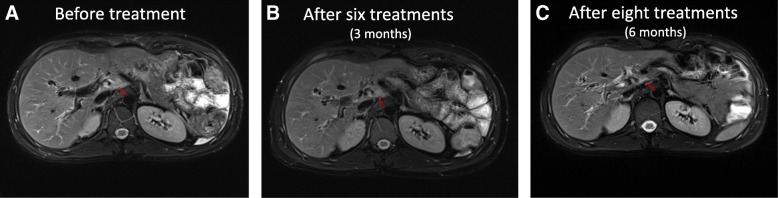
Upper abdominal MRI examinations were taken at 3 time points, **a** 2 weeks before treatment, **b** 3 month’s clinical effect post treatment and **c** 6 months clinical effect post treatment. In this figure, we show representative MRI images obtained before entry into the clinical trial and after the 8th treatment course

**Fig. 4:**
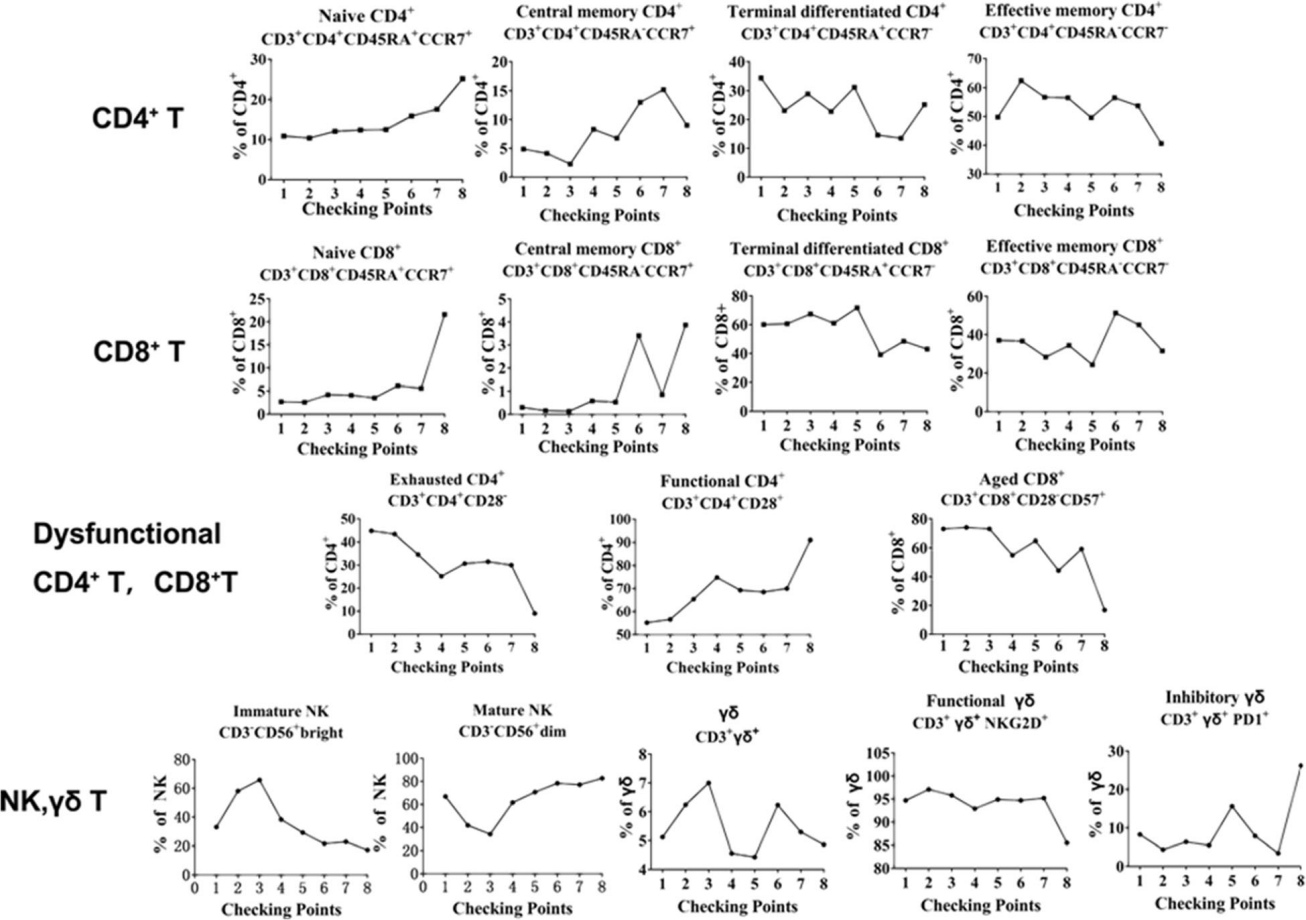
The changes in immunophenotyping before (‘1’) and after (‘2’ - ‘8’) γδ T cell treatments. The results showed that γδ T cell therapy could greatly improve immunity by regulating the immunological functions of peripheral immune cells, as the administration of γδ T cells was associated with an increase of the functional CD3 + CD4 + CD28+ T cells and CD3 + CD8 + CD28+ T cells, and with a decrease of CD3 + CD4 + CD28- T cells and CD3 + CD4 + CD28-CD57+ T cells. In these graphs, checking point ‘1’ means immunophenotyping without γδ T cell treatment, while checking points ‘2’ - ‘8’ stand for immunophenotyping from the first time to the seventh γδ T cell treatments

Firstly, from the MRI images (Fig. 3), we can see that the size of the lymph nodes is markedly reduced, visualizing that lymph nodes metastases of the patient were gradually eliminated with increasing infusion times of Vδ2 T cells. Such visual images indicated that the patient greatly benefited from allogenic Vγ9Vδ2 T cell treatment in this case. Then, the immunophenotypes of the patient before and after γδ T cell treatment were analyzed (Fig. 4). We evaluated immunophenotype variations of CD4+, CD8+, NK, and γδ T cells using immunofluorescence labeling and flow cytometry. The results showed that γδ T cell therapy could greatly improve immunity by regulating the immunological functions of these immune cells, as the administration of γδ T cells was associated with an increase of the functional CD3 + CD4 + CD28+ T cells and CD3 + CD8 + CD28+ T cells, and decrease of CD3 + CD4 + CD28- T cells and CD3 + CD4 + CD28-CD57+ T cells. It should be mentioned that, the patient was oraling Rapamune 2 mg, Ursofalk 500 mg once a day, these two drugs serve as anti-transplanet rejection since the patient received liver transplantation.

**Fig. 5:**
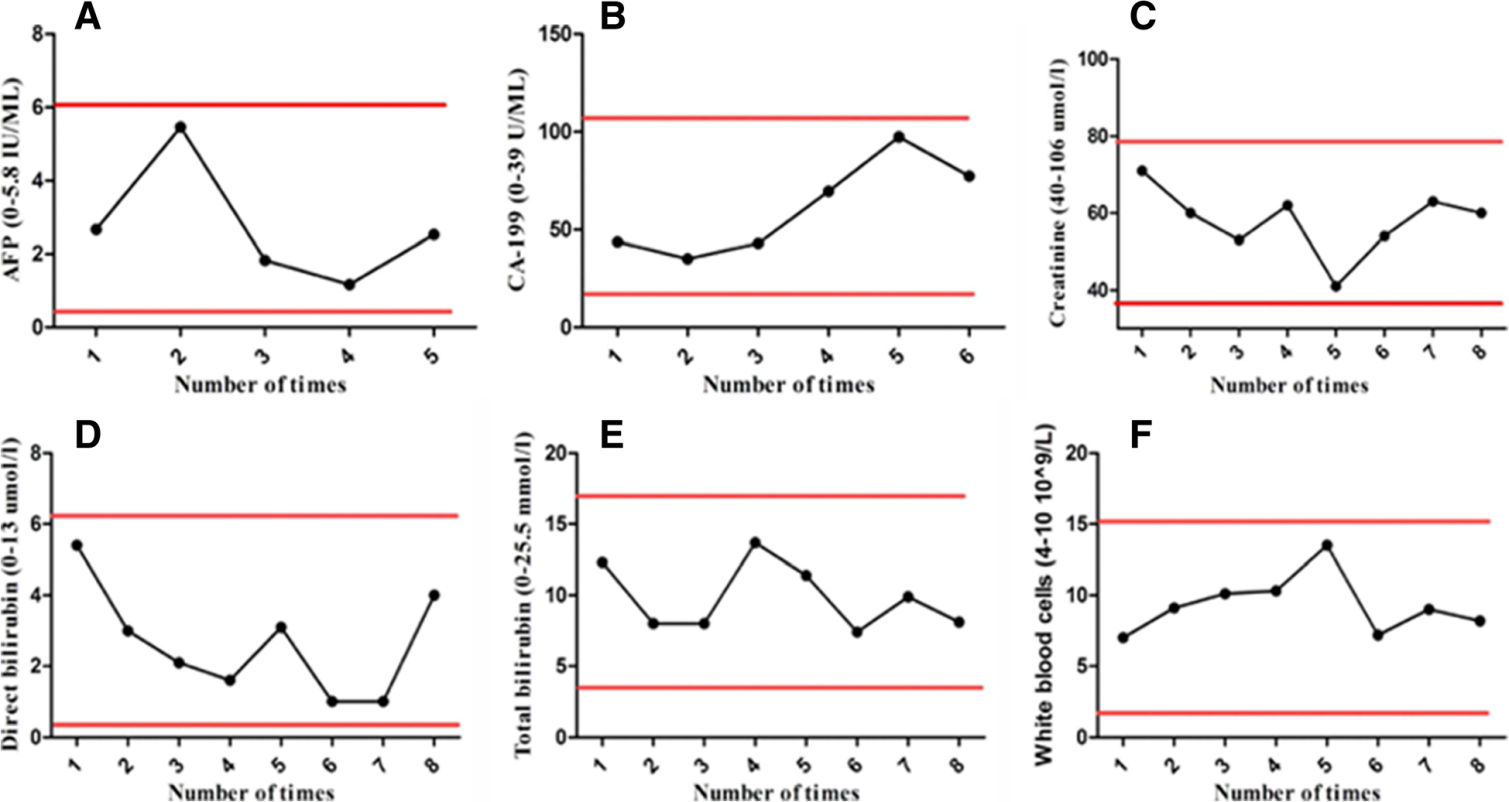
Blood biochemical examination. All biochemical markers maintained at a low lever before and after γδ T cell treatments, showing no difference in the level of **a** alpha-fetoprotein (AFP), **b** carbohydrate antigen (CA-199), **c** serum Creatinine, **d** serum direct bilirubin, **e** Serum total bilirubin, and **f** total white blood cells

Biochemical examination results clearly demonstrated that allogenic Vγ9Vδ2 T cells were safe for immunotherapeutic application (Fig. 5). We noticed that the expression of tumor marker molecule was maintained at a low level during γδ T cell treatment, and there was no liver function impairment. This was consistent with the stable physical condition and sound prognosis of the patient. Altogether, this clinical trial study clearly evidenced that there were no observed complications related to γδ T cell infusion.

## Discussion

Because γδ T cells can bridge the gap between innate and adaptive immune systems and are critical in surveillance and defense of tumorigenesis and infection, γδ T cell-based immunotherapy could be developed into a promising treatment for tumor control or elimination [24–30], particularly for diseases refractory to traditional treatments (surgery, chemotherapy and radiotherapy). It’s known that γδ T cells can recognize target cells (cancer cells or pathogen-infected cells) in a MHC independent way, which implicates with the immunological mechanism of high allogeneic safety of γδ T cells [31]. This clinical trial study also clearly evidenced that there were no observed complications related to γδ T cell infusion.

In this report, we evaluated the safety and efficacy of allogenic Vγ9Vδ2 T cells for the first time as a new type of immunotherapy to treat a patient (stage IV Cholangiocarcinoma and liver transplanted) with recurrent mediastinal lymph node metastasis. The patient received γδ T cell treatment every 2 weeks for the first six treatments, and every 4 weeks for the last two treatments (between August, 2017 and February, 2018) (Fig. 2). Clinical results clearly demonstrated that allogenic Vγ9Vδ2 T cells were safe for immunotherapeutic application, and that allogenic Vγ9Vδ2 T cell treatment eliminated tumor metastases in this case (Fig. 3). Firstly, from the MRI images (Fig. 3), we can see that the size of the lymph nodes is markedly reduced, visualizing that lymph nodes metastases of the patient were gradually eliminated with increased infusion times of Vδ2 T cells. Then, the immunophenotypes of the patient before and after γδ T cell treatment were analyzed (Fig. 4). We evaluated immunophenotypes of CD4+, CD8+, NK, and γδ T cells using immunofluorescence labeling and flow cytometry. We found that γδ T cell therapy could greatly improve immunity by regulating αβ T cells and NK cells. For instance, it could elevate ratio of naïve, functional CD4+, and CD8+ T cells, and reduce exhausted and aged CD4+, CD8+ T cells, and so on (Fig. 4). Previous literatures [21, 26, 32, 33] proposed that γδ T cells can regulate other immune cells including potentiating functions of CD4+, CD8+ T cells, maturing dendritic cells and activate neutrophils. As a further step, our work here revealed that Vδ2 subpopulation transfer therapy can affect αβ T cell differentiation and NK maturation, particularly, for example, by reducing exhausted and aged αβ T cells and elevating functional αβ T cells. Additionally, according to Fig. 5, we noticed that the expression of tumor marker molecules AFP and CA-199 was maintained at a low lever during γδ T cell treatment, with no observed impaired liver functions. This is consistent with the stable physical condition and sound prognosis of the patient.

In conclusion, in this case report, we conducted allogenic γδ T cell immunotherapy of Cholangiocarcinoma for the first time. The clinical outcome evidenced that allogenic γδ T cell therapy was very safe and displayed reliable efficacy in liver cancer treatment. This exciting trial opened a new window for cancer immunotherapy and could inspire more clinical trial studies, based upon allogenic γδ T cell. Allogenic γδ T cells could be developed into a very promising ‘immune drug’ for malignant tumor therapy. Our report will undoubtedly represent the next frontier for immunotherapeutic innovations in cancer research and treatment.

## Additional file

Additional file 1: **Figure S1.** Purity of infused allogenic Vγ9Vδ2 T cells of all 8 treatment courses is > 85%. According to flow cytometry data, rest non Vδ2 T cells including Vδ1 T cells, NK cells, B cells, NKT cells, CD8^+^T cells, CD4^+^T cells, CD4^+^CD8^+^T cells, and CD4^-^CD8^-^T cells. **Figure S2.** Molecular phenotypes of allogenic Vγ9Vδ2 T cells cultured using our developed specific culture formula, showing high expression of killing related molecules (like NKG2D, IFN-γ, TNF-α, CD107a) and low expression of inhibitory molecules like PD-1. (PPTX 255 kb)

## Abbreviations

CCA: Cholangiocarcinoma
I ^125^: Iodine-125
PBMCs: Peripheral blood mononuclear cells
PSC: Primary Sclerosing Cholangitis
γδ: Gamma delta
