# Supplementary material for: Allogenic Vγ9Vδ2 T cell as new potential immunotherapy drug for solid tumor: a case study for cholangiocarcinoma
Source: J Immunother Cancer. 2019 Feb 8;7:36. doi: 10.1186/s40425-019-0501-8 (PMC6368763; doi:10.1186/s40425-019-0501-8)
Supplement: Supplementary file 1 — Figure S1. Purity of infused allogenic Vγ9Vδ2 T cells of all 8 treatment courses is > 85%. According to flow cytometry data, rest non Vδ2 T cells including Vδ1 T cells, NK cells, B cells, NKT cells, CD8+T cells, CD4+T cells, CD4+CD8+T cells, and CD4-CD8-T cells. Figure S2. Molecular phenotypes of allogenic Vγ9Vδ2 T cells cultured using our developed specific culture formula, showing high expression of killing related molecules (like NKG2D, IFN-γ, TNF-α, CD107a) and low expression of inhibitory molecules like PD-1. (PPTX 255 kb) [file 40425_2019_501_MOESM1_ESM.pptx]

## Slide 1
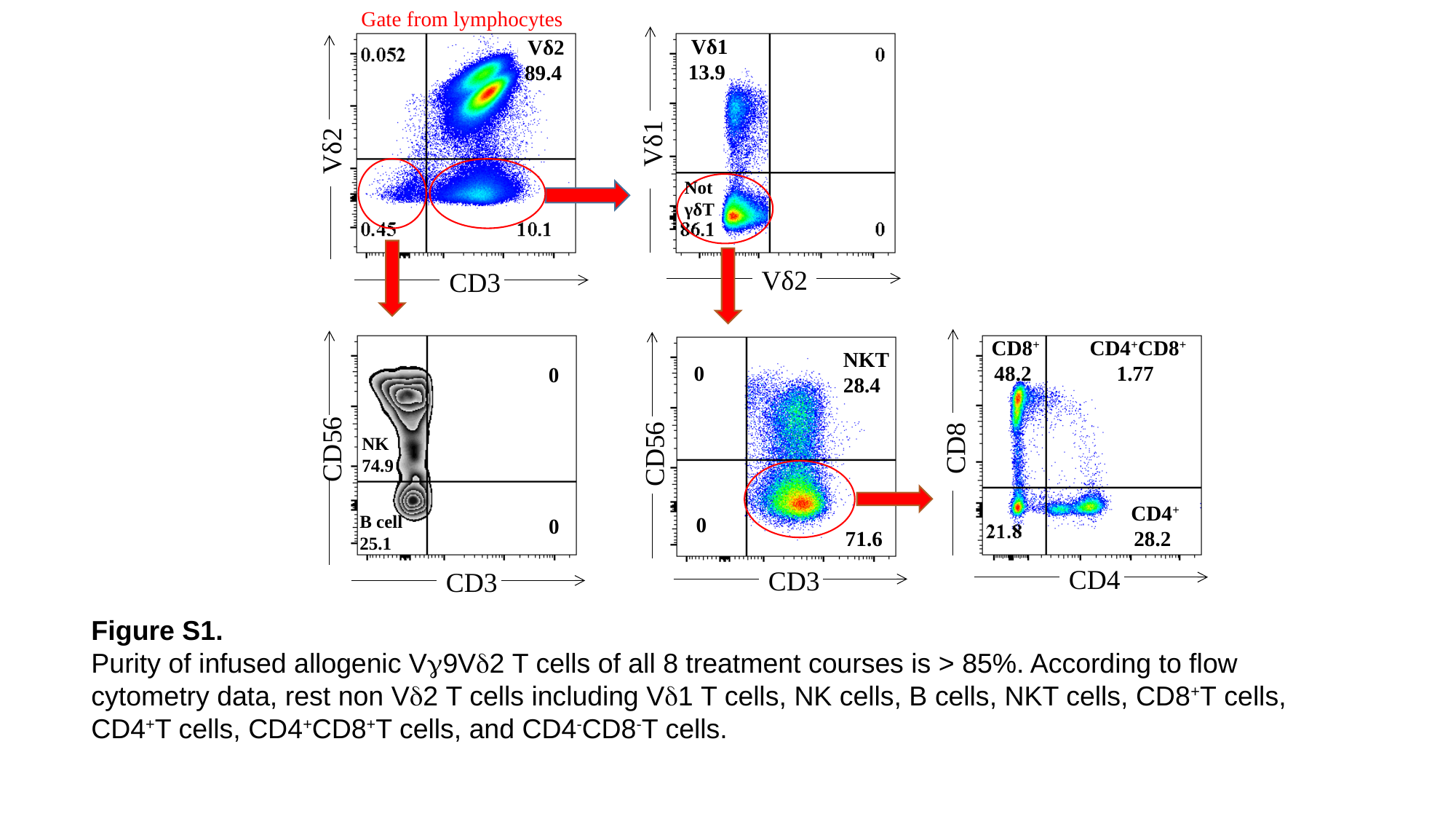

Gate from lymphocytes
Vδ2
CD3
Vδ2
89.4
Vδ1
Vδ2
Vδ1
13.9
Not
γδT
CD56
CD3
NK
74.9
B cell
25.1
0
0
CD8
CD4
CD8+
48.2
CD4+
28.2
CD56
CD3
NKT
28.4
0
0
71.6
CD4+CD8+
1.77
Figure S1.
Purity of infused allogenic Vγ9Vδ2 T cells of all 8 treatment courses is > 85%. According to flow cytometry data, rest non Vδ2 T cells including Vδ1 T cells, NK cells, B cells, NKT cells, CD8+T cells, CD4+T cells, CD4+CD8+T cells, and CD4-CD8-T cells.

## Slide 2
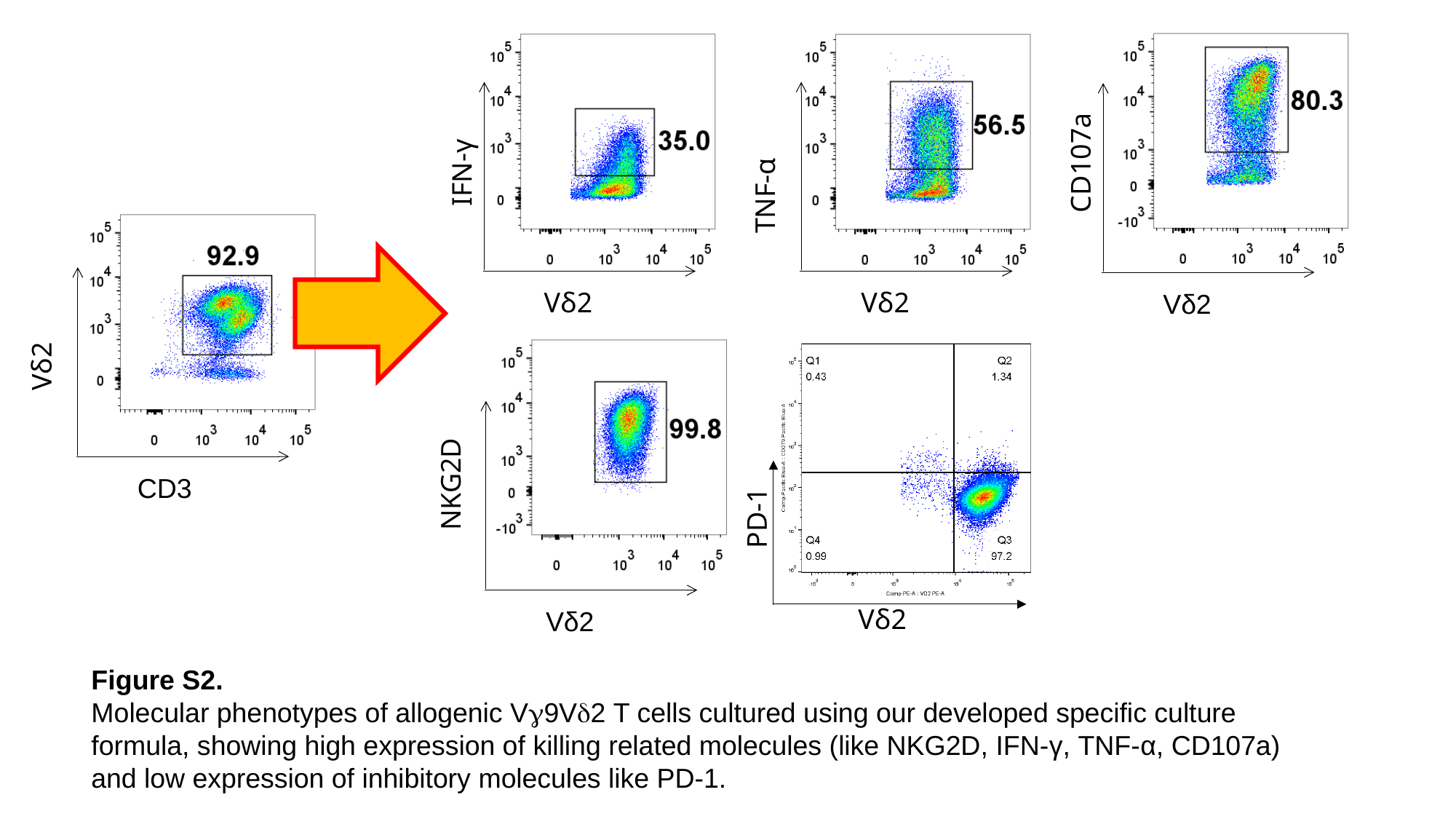

CD107a
Vδ2
IFN-γ
Vδ2
TNF-α
Vδ2
Vδ2
CD3
NKG2D
Vδ2
PD-1
Vδ2
Figure S2.
Molecular phenotypes of allogenic Vγ9Vδ2 T cells cultured using our developed specific culture formula, showing high expression of killing related molecules (like NKG2D, IFN-γ, TNF-α, CD107a) and low expression of inhibitory molecules like PD-1.
